# Use of pressure muscle index to predict the contribution of patient’s inspiratory effort during pressure support ventilation: a prospective physiological study

**DOI:** 10.3389/fmed.2024.1390878

**Published:** 2024-04-26

**Authors:** Ran Gao, Jian-Xin Zhou, Yan-Lin Yang, Shan-Shan Xu, Yi-Min Zhou, Linlin Zhang, Ming-Yue Miao

**Affiliations:** ^1^Department of Critical Care Medicine, Beijing Tiantan Hospital, Capital Medical University, Beijing, China; ^2^Clinical and Research Center on Acute Lung Injury, Emergency, and Critical Care Center, Beijing Shijitan Hospital, Capital Medical University, Beijing, China

**Keywords:** pressure support ventilation, muscle pressure index, inspiratory effort, respiratory mechanics, protective ventilation

## Abstract

**Background:**

The successful implementation of assisted ventilation depends on matching the patient’s effort with the ventilator support. Pressure muscle index (PMI), an airway pressure based measurement, has been used as noninvasive monitoring to assess the patient’s inspiratory effort. The authors aimed to evaluate the feasibility of pressure support adjustment according to the PMI target and the diagnostic performance of PMI to predict the contribution of the patient’s effort during ventilator support.

**Methods:**

In this prospective physiological study, 22 adult patients undergoing pressure support ventilation were enrolled. After an end-inspiratory airway occlusion, airway pressure reached a plateau, and the magnitude of change in plateau from peak airway pressure was defined as PMI. Pressure support was adjusted to obtain the PMI which was closest to −1, 0, +1, +2, and + 3 cm H_2_O. Each pressure support level was maintained for 20 min. Esophageal pressure was monitored. Pressure–time products of respiratory muscle and ventilator insufflation were measured, and the fraction of pressure generated by the patient was calculated to represent the contribution of the patient’s inspiratory effort.

**Results:**

A total of 105 datasets were collected at different PMI-targeted pressure support levels. The differences in PMI between the target and the obtained value were all within ±1 cm H_2_O. As targeted PMI increased, pressure support settings decreased significantly from a median (interquartile range) of 11 (10–12) to 5 (4–6) cm H_2_O (*p* < 0.001), which resulted in a significant increase in pressure–time products of respiratory muscle [from 2.9 (2.1–5.0) to 6.8 (5.3–8.1) cm H_2_O•s] and the fraction of pressure generated by the patient [from 25% (19–31%) to 72% (62–87%)] (*p* < 0.001). The area under receiver operating characteristic curves for PMI to predict 30 and 70% contribution of patient’s effort were 0.93 and 0.95, respectively. High sensitivity (all 1.00), specificity (0.86 and 0.78), and negative predictive value (all 1.00), but low positive predictive value (0.61 and 0.43) were obtained to predict either high or low contribution of patient’s effort.

**Conclusion:**

Our results preliminarily suggested the feasibility of pressure support adjustment according to the PMI target from the ventilator screen. PMI could reliably predict the high and low contribution of a patient’s effort during assisted ventilation.

**Clinical trial registration:** ClinicalTrials.gov, identifier NCT05970393.

## 1 Introduction

The successful implementation of pressure support ventilation (PSV), one of the most commonly used assisted ventilatory modes, depends on matching the need of the patient’s inspiratory demand with the ventilator support (1–3). Recent studies have implied that under- and over-assistance, which may induce high and low patient’ inspiratory efforts, are potentially associated with lung- and diaphragm-injury (4, 5). It is of clinical significance to evaluate the patient’s inspiratory effort and its contribution to determining the degree of support following the patient’s condition.

During assisted ventilation, the patient’s inspiratory effort can be evaluated by the measurement of esophageal pressure, but it is often used for research purposes because of its complexity in monitoring procedures and parameter calculations (6, 7). Alternatively, the setting of pressure support according to tidal volume and respiratory rate is clinically employed to achieve a stable breathing pattern and a normal range of arterial blood gases (8). However, studies have shown that over-assistance may not be uncommon with this pressure support setting principle (9, 10). In clinical practice, the overlook of over-assistance is mainly due to a lack of valid and simple monitoring.

During PSV, a plateau airway pressure can be induced by end-inspiratory occlusion (11). The difference between the peak and plateau airway pressure is defined as the pressure muscle index (PMI), which was first introduced as an inspiratory effort assessment parameter by Foti and coworkers in 1997 (12). The major advantage of PMI measurement is its accessibility from the ventilator screen without the need for other equipment, albeit no study has demonstrated the accuracy of PMI measurement obtained from the ventilator screen. Although studies have shown that PMI closely correlates with esophageal pressure-derived effort variables (13, 14) and can reliably detect low and high inspiratory efforts (14), no study has been conducted to evaluate the contribution of the patient’s effort during PSV, which could provide basic data for further investigation on the utility of PMI as an indicator for pressure support adjustment.

In the present study, mechanically ventilated patients undergoing PSV were enrolled, and the feasibility of a pressure support adjustment algorithm according to the PMI measurement on ventilator screen was evaluated. Esophageal pressure was monitored, and the fraction of pressure generated by the patient was calculated. The objective was to establish the range of PMI values to indicate the different contributions of the patient’s inspiratory effort during PSV.

## 2 Materials and methods

This prospective physiological study was conducted in the ICU at Beijing Tiantan Hospital, Capital Medical University. The study protocol was approved by the Institutional Review Board of the hospital (KY-2023-001-02) and registered at ClinicalTrials.gov (NCT05970393) on August 1, 2023, by Jian-Xin Zhou. Written informed consent was obtained from the patients or their legal representatives. The study design, performance, and report were compliant with the Standards for Reporting of Diagnostic Accuracy (STARD) guidelines (15).

### 2.1 Patients and routine practice for PSV

Mechanically ventilated patients were consecutively screened daily and enrolled within 24 h after switching to PSV mode. Exclusion criteria included: (1) age younger than 18 years; (2) esophageal or gastric tumor, trauma, or surgery; (3) pneumothorax or lung surgery; (4) diaphragm injury or dysfunction; (5) neuromuscular diseases; (6) brain stem lesions with abnormal respiratory drive presented with unstable respiratory rhythm and amplitude; (7) pregnancy, or (8) anticipating withdrawal of life support.

For all enrolled patients, standard clinical care for mechanical ventilation is performed following the local clinical guidelines, except for pressure support adjustment during the study procedure.

The nurse-to-bed ratio in the ICU is 2.5:1. Analgesia is routinely used in mechanically ventilated patients with continuous infusion of fentanyl or remifentanil. Sedation with propofol or dexmedetomidine is used when the patient exhibits agitation and a light sedation level is maintained (Riker’s Sedation-Agitation Scale of 3 to 4). During the study procedure of pressure support setting, analgesia and sedation were not adjusted.

In our routine clinical practice, the initial PSV settings are determined by the responsible attending physician according to the principles including (16): (1) pressure support is adjusted to obtain a tidal volume between 6 and 8 mL/kg predicted body weight and respiratory rate below 30 breaths/min; (2) the inspiratory trigger sensitivity is set as 1–2 L/min for flow-trigger or 1.5–3 cm H_2_O for pressure-trigger; (3) inspiration-to-expiration cycle-off is set as 25% of the peak inspiratory flow; and (4) the fraction of inspired oxygen (FiO_2_) and positive expiratory end pressure (PEEP) are set to maintain pulse arterial oxygen saturation (SpO_2_) between 90 and 95%. Specifically, if SpO_2_ is lower than 90%, PEEP and then FiO_2_ will be increased by 2 cm H_2_O and 0.1; whereas if SpO_2_ is higher than 95%, FiO_2_ and then PEEP will be decreased by 0.1 and 2 cm H_2_O.

### 2.2 Study protocol

#### 2.2.1 Esophageal pressure monitoring

After enrollment, an esophageal balloon catheter (Cooper catheter: LOT 177405, Cooper Surgical, United States) was placed as the method described previously (6, 7). Baydur’s occlusion test was performed to confirm the proper balloon position (17). Two KT 100D-2 pressure transducers (KleisTEK Engineering, Bari, Italy) were connected proximally to the endotracheal tube to measure the airway pressure and the balloon lumen of the esophageal catheter to measure the esophageal pressure, respectively. A heated Fleisch pneumotachograph (Vitalograph Inc., Lenexa, KS, United States) was placed between the Y-piece of the ventilator circuit and the endotracheal tube to measure the flow. Pressure transducers and Fleisch pneumotachograph were connected to an ICU-Lab pressure box (KleisTEK Engineering, Bari, Italy) by 80 cm rigid tube lines. Before each patient’s monitoring, the pressure transducer was calibrated by a water column, and the pneumotachograph with a 1-L calibration syringe (Hans Rudolph, Inc. Shawnee, KS, United States). Flow, airway pressure, and esophageal pressure signals were displayed continuously and saved (ICU-Lab 2.6 Software Package, KleisTEK Engineering, Bari, Italy) on a laptop for offline analysis, at a sample rate of 200 Hz.

#### 2.2.2 Adjustment of pressure support according to PMI measurement on the ventilator screen

Dräger V500 (Dräger, Lubeck, Germany) ventilator was used in the present study. During PSV, an end-inspiratory airway occlusion was performed to induce a plateau airway pressure. Airway occlusion was repeated until the plateau pressure met the readable criteria recommended by the previous report, i.e., steep ramp of plateau shorter than 800 msec and total duration of plateau longer than 2 s with an airway pressure fluctuation less than 0.6 cm H_2_O/s (18). PMI was measured on the ventilator screen using the freeze and sliding crusher as shown in the Supplemental Digital Content (Supplementary Video S1). PMI was defined as the peak airway pressure (just before the end-inspiratory occlusion indicated by the onset of zero-flow) minus the plateau airway pressure (1 s after the occlusion) (11, 12).

An algorithm of pressure support adjustment according to PMI measurement on the ventilator screen was designed based on our previous study (14). First, pressure support was set at 20 cm H_2_O and PMI was measured. Usually, PMI was a negative value at this pressure support level. Thereafter, pressure support was downward adjusted to obtain the PMI which was closest to −1, 0, +1, +2, and + 3 cm H_2_O. The other ventilator settings remained unchanged during adjustment. Each pressure support level was maintained for 20 min.

During the study, the measurement of PMI on the ventilator screen and adjustment of pressure support was performed by one investigator (RG) who was trained before the start of the study. During the training, several key points were emphasized following our experience and previous studies including (14, 18, 19):

1. Check the air leak before each PMI measurement, including cuff check and observation of inspiratory and expiratory tidal volume difference;
2. Observe the flow-time waveform during occlusion (maintaining zero flow);
3. A longer than a 2-s duration of end-inspiratory occlusion;
4. Observe the plateau airway pressure during the end-inspiratory occlusion (flat shape).

At the end of each 20-min equilibration period, one end-expiratory occlusion was performed with only one inspiratory effort induced by the occlusion. After 60 s, one end-inspiratory occlusion was performed as described above. After airway occlusions, parameters of inspiratory effort were collected for offline analysis.

Maintenance of each pressure support level was stopped when the patient showed the following signs: (1) respiratory distress including respiratory rate above 30 breaths/min, the use of accessory respiratory muscles, diaphoresis, agitation, and the appearance of abdominal or thoracic paradoxical movements; (2) SpO_2_ lower than 90%; (3) cardiac arrhythmia; and (4) unstable hemodynamics including hypotension (systolic blood pressure lower than 90 mm Hg) or hypertension (systolic blood pressure higher than 160 mm Hg). The study procedure was also stopped if the patient exhibited apnea as indicated by the initiation of backup ventilation during PSV.

### 2.3 Data collection and measurements

**Figure 1:**
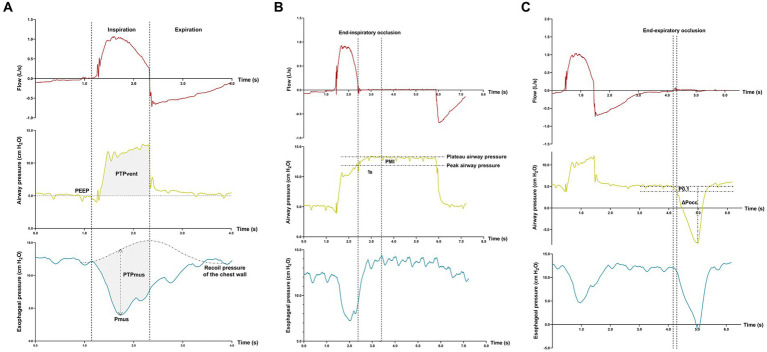
Offline analyses of inspiratory effort. Flow-, airway pressure-, and esophageal pressure–time tracings are displayed. Measurements of the pressure generated by respiratory muscle during inspiration (Pmus), pressure–time-product of ventilator insufflation (PTPvent), and pressure–time-product of respiratory muscle (PTPmus) **(A)**; Measurement of pressure muscle index (PMI) induced by an end-inspiratory airway occlusion **(B)**; Measurements of the negative airway pressure generated during the first 100 ms (P0.1) and the maximal negative swing of airway pressure (∆Pocc) against an end-expiratory airway occlusion **(C)**.

Offline analyses of airway pressure- and esophageal pressure–time waveforms, which are schematically shown in Figure 1, were performed independently by two investigators (SSX and YMZ). When the two measurements were discrepant, a group discussion was held with two other senior investigators (YLY and JXZ) to reach a consensus.

#### 2.3.1 Inspiratory effort parameters derived from esophageal pressure monitoring

The pressure generated by respiratory muscle during inspiration (Pmus) and pressure–time-product (PTP) of respiratory muscle (PTPmus) were calculated according to previous reports (6, 7). Breathings without esophageal pressure artefacts and patient-ventilator asynchrony were identified within the last 3 min in each equilibration period of pressure support level, and measurements were averaged.

Pmus was calculated as the maximal difference between the esophageal pressure and recoil pressure of the chest wall which was constructed by the estimation of 4% of the predicted value of vital capacity (Figure 1A) (20). PTPmus per breath was calculated as the time-integral of the Pmus, from the onset of inspiratory effort to the end of ventilator insufflation.

In the present study, Pmus was used as the reference for inspiratory effort measurement. The low and high effort was pre-defined as Pmus <5 and > 10 cm H_2_O, respectively (21–23).

PTP of ventilator insufflation (PTPvent) per breath was obtained as the area between the airway pressure and the set PEEP during inspiration (Figure 1A). The fraction of pressure generated by the patient per breath (PTPratio) during PSV was calculated as the following equation (24):


$${PTP}_{\mathrm{ratio}}=\frac{\mathrm{PTPmus}}{\mathrm{PTPmus}+\mathrm{PTPvent}}$$

#### 2.3.2 Inspiratory effort parameters derived from airway pressure–time tracings

Inspiratory effort parameters derived from airway pressure–time tracings were measured from the end-expiratory and end-inspiratory airway occlusion maneuvers.

After the onset of end-inspiratory airway occlusion, the airway pressure reached a plateau, and PMI was measured as the difference between the peak and plateau airway pressure (Figure 1B) (12–14). Against an end-expiratory airway occlusion, the negative airway pressure generated during the first 100 msec (airway occlusion pressure, P0.1) (25, 26) and the maximal negative swing of airway pressure (∆Pocc) (21, 27) were measured (Figure 1C).

### 2.4 Statistical analysis

Continuous variables are expressed as the median and interquartile range (IQR) and were compared among different PMI-targeted pressure support levels using the Friedman’s nonparametric test, followed by pairwise post-hoc analysis with the Wilcoxon test. The difference in PMI between the obtained and at the end of the 20-min equilibration period was compared using a paired Wilcoxon test.

Linear mixed-effects regression models were used to analyze the association of PMI with Pmus, PTPmus, and PTPratio, with patients as a random effect and PMI groups as repeated measures.

Using the receiver operating characteristics (ROC) curve analysis, the diagnostic performance was evaluated for PMI, and traditional parameters for pressure support adjustment including tidal volume and respiratory rate, to predict PTPratio higher than 70% or lower than 30%, as well as to detect high and low inspiratory effort. The area under the curve (AUC) and 95% confidence interval (CI) were calculated, and the best cutoff values were identified by the Youden index. A comparison of AUC was performed using the DeLong test. Sensitivity, specificity, and positive and negative predictive values (PPV and NPV) were calculated using the standard formula.

In the present study, we are particularly interested in the predictive performance of PMI for high (>70% PTPratio) and low (<30% PTPratio) contribution of the patient’s inspiratory effort during PSV. Because the distribution of PTPratio has not been well described, we did not perform formal sample size estimation but planned to collect 100 levels of PMI-targeted pressure support settings, namely 20 patients each with adjustments of five pressure support levels. Additional patients were enrolled if tested pressure support levels were stopped due to predefined criteria (see above).

Statistical analyses were performed using R (R4.2.0, R Foundation for Statistical Computing, Vienna, Austria), MedCalc (2022 MedCalc Software Ltd., Belgium), and Prism 9 (version 9.1.2, GraphPad Software, United States). A *p*-value less than 0.05 was considered a significant difference.

## 3 Results

Twenty-two patients were enrolled and their clinical characteristics are shown in Table 1. A stable plateau airway pressure was obtained by end-inspiratory occlusion in all patients at all tested pressure support levels. Respiratory distress occurred at the pressure support level targeting a PMI of +3 cm H_2_O in two patients, and the study procedure was stopped. In the other three patients, adjustment of pressure support could not obtain a PMI of +2 cm H_2_O (could only obtain a PMI of +1 or + 3 cm H_2_O). Therefore, 105 datasets with different PMI-targeting pressure support levels were collected and analyzed.

**Table 1:** Baseline characteristics.

Patient characteristic	n = 22
Age, yr	57 (53, 68)
Male, *n* (%)	16 (73)
Body mass index, kg/m^2^	24.7 (23.4, 26.8)
Main diagnosis, *n* (%)	
Postoperative	12 (55)
Acute respiratory failure	6 (27)
Sepsis	3 (14)
Trauma	1 (4)
Reasons for mechanical ventilation	
Acute hypoxemic respiratory failure	16 (73)
Airway protection	6 (27)
Acute Physiology and chronic health evaluation II	15 (10, 23)
Mechanical ventilation days before inclusion	3.5 (1.8, 6.3)
PSV settings at enrollment	
Pressure support, cm H_2_O	8 (6, 8)
PEEP, cm H_2_O	5 (5, 5)
FiO_2_	0.40 (0.39, 0.40)
Blood gas at enrollment	
PaO_2_, mm Hg	106 (86, 133)
PaO_2_/FiO_2_	266 (223, 331)
PaCO_2_, mm Hg	39 (38, 44)
Inspiratory effort at enrollment	
Respiratory rate, breaths/min	16 (13, 20)
Tidal volume, ml/kg predicted body weight	6.9 (6.3, 8.4)
Pmus, ml/cm H_2_O	5.9 (4.1, 7.7)
PTPmus, cm H_2_O⋅s⋅min^−1^	4.0 (3.2, 6.3)
PTPratio, %	42 (35, 59)
PMI, cm H_2_O	1.2 (0.5, 2.0)
P0.1, cm H_2_O	1.2 (0.6, 1.8)
∆Pocc, cm H_2_O	7.3 (5.9, 11.6)
Analgesia and sedation at enrollment	
Use of analgesics, *n* (%)	17 (77%)
Use of sedatives, *n* (%)	9 (41%)
Sedation and Agitation Score	3 (3, 4)

Data are present as median (interquartile range).

FiO_2_, fraction of inspired oxygen; PaCO_2_, arterial partial pressure of carbon dioxide; PaO_2_, arterial partial pressure of oxygen; PEEP, positive end-expiratory pressure; PMI, pressure muscle index; Pmus, pressure generated by respiratory muscle during inspiration; PSV, pressure support ventilation; PTPmus, pressure–time-product of respiratory muscle; P0.1, airway occlusion pressure; ΔPocc, maximal negative swing of airway pressure against end-expiratory occlusion.

**Figure 2:**
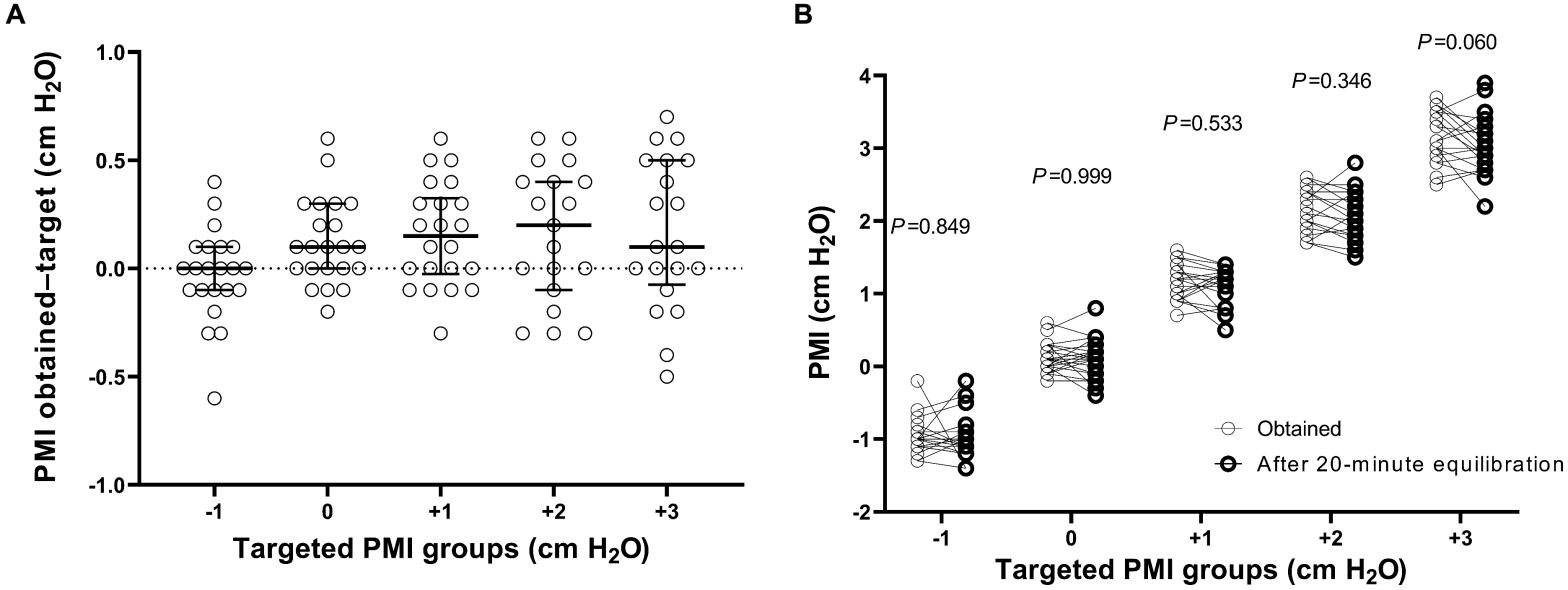
The difference in pressure muscle index (PMI) between the target and obtained values **(A)**, and PMI between the obtained and at the end of the 20-min equilibration period **(B)**. *p* values shown in figure indicate the statistical significance in PMI between the obtained and at the end of the 20-min equilibration period at different targeted PMI levels by using a paired Wilcoxon test.

The differences in targeted and obtained PMI values were all within ±1 cm H_2_O (Figure 2A). No significant difference was found in PMI between the obtained and at the end of the 20-min equilibration period (*p* > 0.05, Figure 2B).

### 3.1 Comparisons among different targeted PMI groups

**Figure 3:**
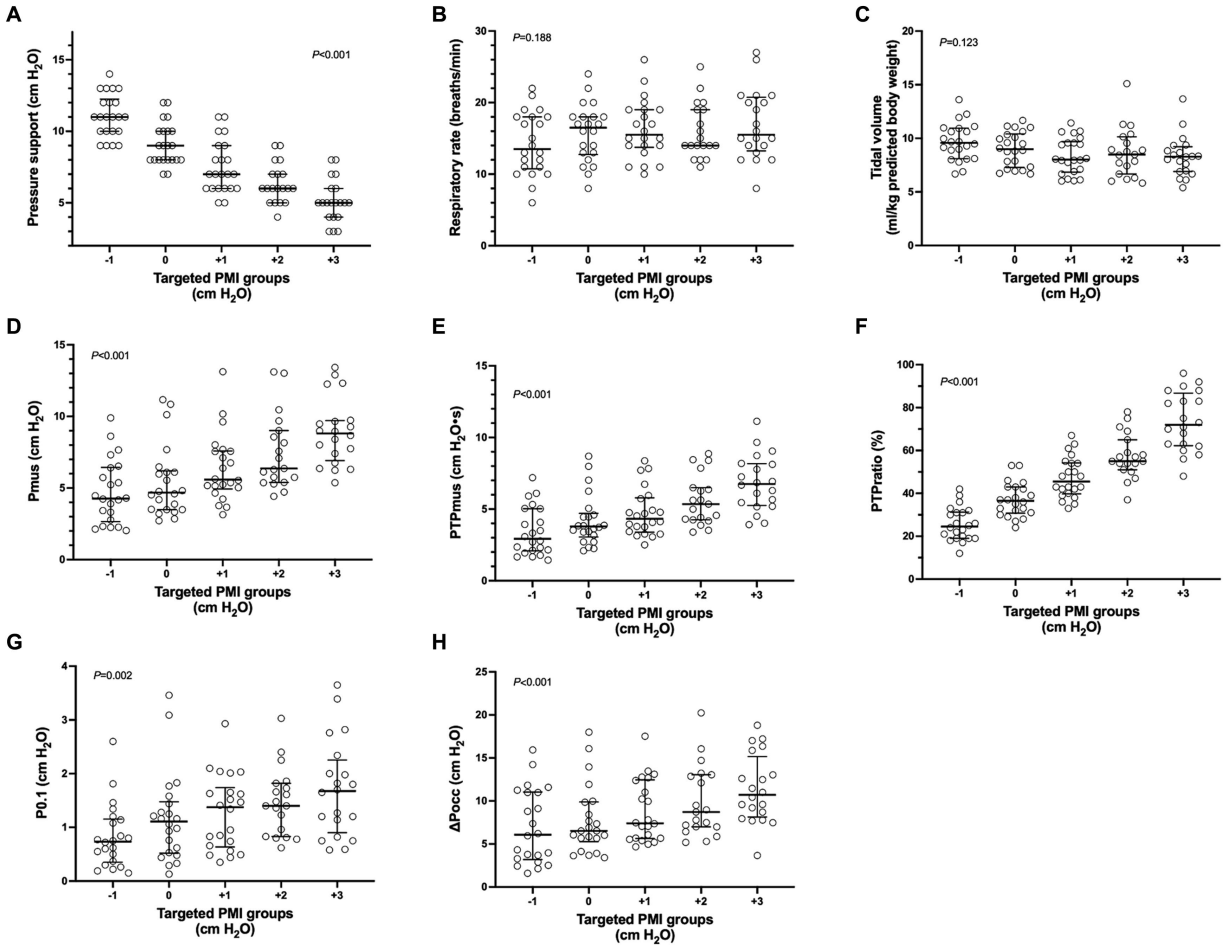
Parameters among different targeted pressure muscle index (PMI) groups. Individual data (circle), median and interquartile range (horizontal line) are shown. Significant differences were found in pairwise comparison among different targeted PMI groups in pressure support **(A)**, pressure generated by respiratory muscle during inspiration (Pmus) **(D)**, pressure–time-product of respiratory muscle (PTPmus) **(E)**, fraction of pressure generated by the patient per breath (PTPratio) **(F)**, negative airway pressure generated during the first 100 msec (P0.1) **(G)** and maximal negative swing of airway pressure (∆Pocc) **(H)** against an end-expiratory airway occlusion (*p* < 0.05). No significant differences were found in respiratory rate **(B)** and tidal volume **(C)**.

Comparisons of parameters among different targeted PMI groups are shown in Figure 3. Although the level of pressure support significantly decreased as targeted PMI increased (*p* < 0.001, Figure 3A), tidal volume and respiratory rate were not significantly different among PMI groups (*p* = 0.123 and 0.188, Figures 3B,C). As PMI increased, Pmus, PTPmus and PTPratio (*p* < 0.001, Figures 3D–F), as well as P0.1 (*p* = 0.002, Figure 3G) and ∆Pocc (*p* < 0.001, Figure 3H) increased significantly.

### 3.2 Association of PMI with Pmus, PTPmus, and PTPratio

Linear mixed-effects regression analysis showed a significant correlation of PMI with Pmus (between-patients *R^2^* = 0.281; within-patients *R^2^* = 0.835; *p* < 0.001), PTPmus (between-patients *R^2^* = 0.367; within-patients *R^2^* = 0.831; *p* < 0.001), and PTPratio (between-patients *R^2^* = 0.535; within-patients *R^2^* = 0.918; *p* < 0.001).

### 3.3 Diagnostic performance of PMI to detect high and low inspiratory effort

Using Pmus as the reference standard, PMI showed discriminative accuracy for detecting high and low inspiratory effort with respective AUC of 0.68 (95%CI: 0.58, 0.77) and 0.82 (95%CI: 0.73, 0.89) (Table 2). No significant differences were found in AUC among PMI, tidal volume and respiratory rate (*p* > 0.05). Sensitivity, specificity, PPV, and NPV are also shown in Table 2.

**Table 2:** Diagnostic performance of pressure muscle index (PMI), tidal volume and respiratory rate to detect low and high inspiratory effort.

References	Parameters	AUC	Cutoff	Sensitivity	Specificity	PPV	NPV
Pmus<5 cm H_2_O	PMI (cm H_2_O)	0.82 (0.73, 0.89)	0.2	0.76 (0.58, 0.89)	0.85 (0.74, 0.92)	0.69 (0.52, 0.84)	0.88 (0.78, 0.94)
	Tidal volume (ml/kg PBW)	0.63 (0.53, 0.73)	9.3	0.79 (0.55, 0.87)	0.49 (0.37, 0.61)	0.41 (0.29, 0.54)	0.83 (0.69, 0.93)
	Respiratory rate (breaths/min)	0.53 (0.43, 0.63)	11	0.94 (0.80, 0.99)	0.18 (0.10, 0.29)	0.34 (0.25, 0.45)	0.87 (0.60, 0.98)
Pmus>10 cm H_2_O	PMI (cm H_2_O)	0.68 (0.58, 0.77)	1.3	0.75 (0.43, 0.95)	0.63 (0.53, 0.73)	0.21 (0.10, 0.36)	0.95 (0.86, 0.99)
	Tidal volume (ml/kg PBW)	0.56 (0.46, 0.66)	7.8	0.92 (0.62, 1.00)	0.33 (0.24, 0.44)	0.15 (0.08, 0.25)	0.97 (0.84, 1.00)
	Respiratory rate (breaths/min)	0.72 (0.62, 0.80)	14	0.92 (0.62, 1.00)	0.63 (0.53, 0.73)	0.24 (0.13, 0.40)	0.98 (0.91, 1.00)

95% confidence intervals are shown in parentheses for diagnostic performance measures.

AUC, area under receiver operating characteristics; PMI, pressure muscle index; PBW, predicted body weight; Pmus, the pressure generated by respiratory muscle during inspiration; PPV, positive predictive value; NPV, negative predictive value.

### 3.4 Diagnostic performance of PMI to predict different contributions of patient’s effort

Table 3 presents the results of diagnostic performance measures for PMI, tidal volume and respiratory rate to predict different contributions of patient’s effort. Using PTPratio higher than 70% and lower than 30% as criteria of high and low patient contribution, PMI showed excellent discriminative accuracy with respective AUC of 0.95 (0.89, 0.98) and 0.93 (0.86, 0.97), and a respective best cutoff value of 0 and 1.8 cm H_2_O. AUC of PMI was significantly higher than those of tidal volume and respiratory rate either for high or low contribution of patient’s effort (*p* < 0.001). High sensitivity (all 1.00), specificity (0.86 and 0.78), and NPV (all 1.00), but low PPV (0.61 and 0.43) were presented for PMI to predict either high or low contribution of patient’s effort (Table 3).

**Table 3:** Diagnostic performance of pressure muscle index, tidal volume and respiratory rate to predict high and low contribution of patient’s inspiratory effort.

References	Parameters	AUC	Cutoff	Sensitivity	Specificity	PPV	NPV
PTPratio<30%	PMI (cm H_2_O)	0.95 (0.89, 0.98)	0.0	1.00 (0.82, 1.00)	0.86 (0.77, 0.93)	0.61 (0.42, 0.78)	1.00 (0.95, 1.00)
	Tidal volume (ml/kg PBW)	0.57 (0.47, 0.67)	7.8	0.89 (0.67, 0.99)	0.33 (0.23,0.44)	0.23 (0.14, 0.34)	0.93 (0.78, 0.99)
	Respiratory rate (breaths/min)	0.55 (0.45, 0.65)	13	0.42 (0.22, 0.67)	0.74 (0.64, 0.83)	0.27 (0.12, 0.46)	0.85 (0.75, 0.92)
Pmus>10 cm H_2_O	PMI (cm H_2_O)	0.93 (0.86, 0.97)	1.8	1.00 (0.78, 1.00)	0.78 (0.68, 0.86)	0.43 (0.27, 0.61)	1.00 (0.95, 1.00)
	Tidal volume (ml/kg PBW)	0.65 (0.55, 0.74)	9.3	0.93 (0.68, 1.00)	0.44 (0.34, 0.55)	0.22 (0.12, 0.34)	0.98 (0.87, 1.00)
	Respiratory rate (breaths/min)	0.58 (0.48, 0.67)	13	0.87 (0.60, 0.98)	0.32 (0.23, 0.43)	0.18 (0.10, 0.28)	0.94 (0.79, 0.99)

95% confidence intervals are shown in parentheses for diagnostic performance measures.

AUC, area under receiver operating characteristics; PMI, pressure muscle index; PBW, predicted body weight; PTPratio, the fraction of pressure generated by the patient; PPV, positive predictive value; NPV, negative predictive value.

## 4 Discussion

In mechanically ventilated patients undergoing PSV, under- and over-assistance may, respectively, induce extremely high and low effort, which have been considered as potential risk factors for ventilator-associated lungs and respiratory muscle injury (4, 5). Therefore, an easily accessible method is required to evaluate inspiratory effort at the bedside. In the present study, we found that pressure support adjustment based on PMI measurements from the ventilator screen was feasible in the majority of conditions. Additionally, PMI demonstrated an excellent discriminative accuracy in predicting either high or low contribution of the patient’s inspiratory effort during PSV, which suggested that PMI might be potentially used as an indicator to provide different degrees of ventilator support.

### 4.1 Accessibility of plateau airway pressure for PMI measurement

During PSV, end-inspiratory occlusion can induce plateau airway pressure, which allows the calculation of static airway driving pressure (plateau airway pressure – PEEP) (11) and PMI (plateau airway pressure – peak airway pressure) (12). However, this maneuver is not widely used in clinical practice mainly due to the possibility of an unstable plateau during the occlusion (18). Several studies reported the accessibility of plateau airway pressure induced by end-inspiratory occlusion. In general, in retrospective analyses of airway pressure waveforms from the original research in which the main purpose was not the measurement of plateau airway pressure, the occurrence of unstable plateau was relatively high (10 to 38%) (13, 19). In one retrospective analysis of 40 patients with 227 measurements during PSV, the absence of expiratory muscle activity could not be excluded in all induced plateau pressure (28). In our previous study, we carried out a training and quality control program, in which several key points were emphasized during the occlusion (check of air leak, observation of zero flow during the occlusion, and a length of occlusion longer than 2 s) (14). Incidence of immeasurable plateau pressure decreased to 8.6%.

In 2022, Bianchi and coworkers systematically evaluated the reliability of occlusion-induced plateau pressure during assisted ventilation. They recommended three criteria for a reliable plateau including a rapid reach of the plateau (< 800 msec), enough duration of the plateau (> 2 s), and a low variation of the plateau (< 0.6 cm H_2_O/s) (18). These criteria are easy to observe on the ventilator screen. In the present study, we used these criteria to guide the performance of end-inspiratory occlusion and thereafter plateau and PMI measurement on the ventilator screen (Supplementary Video S1). Particular attention was paid when performing occlusions at low-pressure support levels since previous data showed that unstable plateau pressure tended to occur in patients with high inspiratory effort which might have resulted from under-assistance (14, 18). By applying this quality control standard, end-inspiratory occlusion induced accessible plateau airway pressure at all pressure support levels in this study, which is comparable to a recently published study employing the same standard (29).

However, it has to be noted that the relatively low proportion of high inspiratory effort in our cohort may have also contributed to the high measurable rate of plateau airway pressure induced by occlusion. Using Pmus higher than 10 cm H_2_O as a reference (21–23), the high inspiratory effort was only found at 11.4% (12/105) of pressure support levels, which was much lower than the results reported by Kyogoku et al. (13). Further investigation is needed on the methods to improve the quality of plateau pressure measurement in patients with high respiratory drive and inspiratory effort.

### 4.2 Feasibility of pressure support adjustment according to PMI target

Up to now, no study has been conducted to evaluate the accuracy of PMI measurement from the ventilator screen. In the present study, we explored the feasibility of pressure support adjustment based on PMI measurements on the ventilator screen. We predefined five PMI targets (−1, 0, +1, +2, and + 3 cm H_2_O) according to our previous data showing cutoff values of low (approximate 0 cm H_2_O) and high (approximate 2 cm H_2_O) inspiratory effort (14). Because pressure support can only be adjusted in 1 cm H_2_O increment by the ventilator, we had to select the PMI value closest to the target when adjusting the pressure support. For example in one patient, we set pressure support as 9 cm H_2_O and obtained a PMI of 1.1 cm H_2_O. After the equilibration of this support level, the next PMI target would be +2 cm H_2_O. Therefore, we decreased pressure support to 8 cm H_2_O, and then the measured PMI was 1.3 cm H_2_O. After we further decreased pressure support to 7 cm H_2_O, the PMI jumped to 2.8 cm H_2_O. Thus, the target PMI of +2 cmH_2_O was not achieved. This situation occurred in three (3/110, 2.7%) pressure support level adjustments. All differences between targeted and obtained PMI values were within ±1 cm H_2_O (Figure 2A), which suggested the feasibility of PMI-directed support adjustment at the bedside.

We also compared the obtained PMI values with the PMI measurement after a 20-min equilibration and found no significant difference (Figure 2B). However, PMI variation over longer time intervals, such as several hours, requires further observation.

### 4.3 Use of PMI to predict the contribution of patient’s effort during PSV

Several studies have shown that PMI is associated with the patient’s inspiratory effort (12–14, 29). In our previous study with 28 patients, we also found that PMI could be used as a reliable monitoring to detect high and low effort at the bedside (14). In the present study with another cohort with 22 patients undergoing PSV, we confirmed these results. Additionally, using PTPratio derived from combined esophageal pressure and airway pressure measurements, we analyzed the contribution of the patient’s inspiratory effort during PSV. PTPratio represents the interaction between the ventilator support and the patient’s effort (24). By titrating pressure support to achieve a PMI from −1 to +3 cm H_2_O, the median (IQR) PTPratio gradually decreased from 72% (62–87%) to 25% (19–31%) (Figure 3F), with a strong association between the two variables (between-patients *R^2^* = 0.535; within-patients *R^2^* = 0.918). These results support the potential use of PMI as an indicator of patient-ventilator interaction in PSV mode.

ROC analysis demonstrated excellent discriminative accuracy (AUC of 0.93 and 0.95) for PMI to predict low (PTPratio lower than 30%) and high (PTPratio higher than 70%) contribution of patient’s effort following the support of pressure, with respective cutoff values of 0 and approximately 2 cm H_2_O (Table 3). For diagnostic performance analysis of both low and high contributions, the high sensitivity (both 1.00 for PTPratio of 30 and 70%) suggested that PMI might help screen unwanted extreme patient’s contribution due to under- and over-assistance support. Meanwhile, the high NPV (also both 1.00) suggested a high probability that PMI could exclude conditions without inappropriate settings.

### 4.4 Clinical implications

Preservation of spontaneous breathing during mechanical ventilation, namely assisted ventilation, can improve gas exchange and lung function, redistribute ventilation and end-expiratory volume to dependent lung areas, and prevent respiratory muscle atrophy (30). However, recent studies showed that, in patients undergoing assisted ventilation, both under- and over-assistance are detrimental (5). Vigorous inspiratory effort due to underlying pathophysiological mechanics or induced by under-assistance may increase lung stress and strain, which is considered the main mechanism of the patient’s self-inflicted lung injury (4). On the other hand, over-assistance or deep sedation may result in decreased respiratory drive and low inspiratory effort, which thereby may induce diaphragm atrophy (5). Extremely high and low effort are also related to diaphragmatic myotrauma due to excessive and insufficient muscle loading, respectively (5). Therefore, monitoring and controlling inspiratory effort is important during assisted ventilation.

Although PSV was initially designed to provide mechanical ventilation during the weaning process in the 1980s, a prospective international cohort study conducted in 2010, which included 927 intensive care units (ICUs) in 40 countries, revealed that PSV had become the most commonly used assisted ventilatory mode during the acute phase of critical illness (1). Some local protocols for mechanical ventilation recommend that PSV, as the first choice of assisted ventilator mode, is initiated when the patient triggers all ventilator breaths during control ventilation (3, 16, 19).

The successful implementation of PSV depends on matching the need of the patient’s inspiratory demand with the ventilator support (3). By adjusting the inspiratory pressure, PSV can provide different degrees of ventilatory support from nearly total alleviation of the work of breathing by the ventilator to just overcoming ventilator accessory dead space during weaning of mechanical ventilation (2). It is of clinical significance to evaluate the patient’s inspiratory effort and its contribution to determine the degree of support following the patient’s condition. However, the inspiratory effort is not routinely measured in clinical practice, and tidal volume and respiratory rate are used as surrogates for the setting of pressure support (8–10). Our results showed that tidal volume and respiratory rate did not change significantly, and the majority of values were not within the target range (6–8 mL/kg of tidal volume and 20–30 breaths/min of respiratory rate) during pressure support titration within the clinically used range (5 to 11 cm H_2_O) (Figures 3A–C). These results suggest that tidal volume and respiratory rate are not sensitive to guide the setting of pressure support. Previous studies have also shown that over-assistance may not be uncommon when pressure support settings rely on tidal volume and respiratory rate (9, 10).

Several non-invasive indices have recently been introduced to assess inspiratory drive and effort during PSV, including P0.1, ∆Pocc and PMI (13, 14, 21, 22, 25, 26). In the present study, we evaluated the diagnostic accuracy of PMI for patient’s effort contribution assessment during PSV. This is the main strength and novelty of the present study, and the results will establish primary data for further research in pressure support adjustment using the PMI.

For PSV mode, most ventilators allow end-inspiratory airway occlusion by a hold function which provides the opportunity to perform the plateau airway pressure measurement. Following the key quality control points we summarized in our center (air leak check, zero flow, duration of occlusion, and shape of plateau) (14), along with the criteria for a readable plateau pressure (a rapid reach of the plateau, enough duration of the plateau, and lower variation of the plateau) recommended by previous investigation (18), PMI measurement is feasible at the bedside.

In the present study, we described an algorithm of pressure support adjustment according to PMI targets. This algorithm may potentially allow ventilator support according to different contributions of the patient’s effort. However, the feasibility of this algorithm in long-term use, such as multiple adjustments during 24 to 48 h, as well as its influence on clinical outcomes, such as duration of mechanical ventilation and weaning process, requires further investigation.

## 5 Limitations

This study has limitations. First, this is a single-centre study with a relatively small sample size. The enrolled patients were relatively stable, which could be indicated by stable oxygenation, low inspiratory effort, and low baseline pressure support level (Table 1). Therefore, our results might not apply to other populations, especially those at risk of high inspiratory effort. There is substantial heterogeneity in the ICU patients and subtypes of patients can have a different response to adjusting mechanical ventilation. For example, patients with severe respiratory failure are quite different from those with relatively healthy lungs (31). Future work is needed to explore how subgroups of patients can have different PMI responses to different pressure support. Second, although PTPratio has been used to indicate the contribution of a patient’s effort during PSV (24), the optimal fraction of patient’s contribution during different periods of mechanical ventilation is still lacking. Third, we did not apply a random setting of pressure support level but a downward adjustment according to PMI targets. The reason for this design was to avoid the possibility of procedure termination due to fatigue at low-pressure support levels. This strategy has been used for the same reason in the previous study (14). Fourth, three consecutive end-inspiratory occlusions were conducted previously to improve the reliability of PMI measurement (12–14). In the present study, we performed a single occlusion and observed the airway pressure waveform on the ventilator screen according to the criteria of readable plateau pressure (18). Our results showed increased accessibility of PMI using this strategy, which is comparable to a recent report (29). However, its applicability in patients with high inspiratory effort needs further investigation.

## 6 Conclusion

In patients undergoing PSV, it is feasible to adjust pressure support according to the PMI target. An excellent discriminative accuracy was found for PMI to predict the high and low contribution of the patient’s inspiratory effort during ventilator support.

## Data availability statement

The original contributions presented in the study are included in the article/Supplementary material, further inquiries can be directed to the corresponding author/s.

## Ethics statement

The studies involving humans were approved by the Institutional Review Board of Beijing Tiantan Hospital, Capital Medical University (KY-2023-001-02). The studies were conducted in accordance with the local legislation and institutional requirements. The participants provided their written informed consent to participate in this study.

## Author contributions

RG: Data curation, Investigation, Project administration, Writing – original draft. J-XZ: Conceptualization, Formal analysis, Funding acquisition, Supervision, Validation, Writing – review & editing. Y-LY: Investigation, Validation, Writing – review & editing. S-SX: Investigation, Writing – review & editing. Y-MZ: Investigation, Validation, Writing – review & editing. LZ: Conceptualization, Writing – review & editing. M-YM: Data curation, Investigation, Validation, Writing – review & editing.

## References

[1] Esteban A Frutos-Vivar F Muriel A Ferguson ND Penuelas O Abraira V. Evolution of mortality over time in patients receiving mechanical ventilation. Am J Respir Crit Care Med. (2013) 188:220–30. doi: 10.1164/rccm.201212-2169OC, PMID: 23631814

[2] Brochard LJ Lellouche F. Pressure support ventilation In: Tobin MJ, editor. Principles and practice of mechanical ventilation. New York: McGraw Hill Companies (2013). 199–227.

[3] Proklou A Karageorgos V Vaporidi K. The potential risks of pressure support ventilation In: Vincent JL, editor. Annual update in intensive care and emergency medicine 2023. Cham: Springer Nature Switzerland AG (2023). 207–20.

[4] Brochard L Slutsky A Pesenti A. Mechanical ventilation to minimize progression of lung injury in acute respiratory failure. Am J Respir Crit Care Med. (2017) 195:438–42. doi: 10.1164/rccm.201605-1081CP, PMID: 27626833

[5] Goligher EC Jonkman AH Dianti J Vaporidi K Beitler JR Patel BK. Clinical strategies for implementing lung and diaphragm-protective ventilation: avoiding insufficient and excessive effort. Intensive Care Med. (2020) 46:2314–26. doi: 10.1007/s00134-020-06288-9, PMID: 33140181 PMC7605467

[6] Akoumianaki E Maggiore SM Valenza F Bellani G Jubran A Loring SH. The application of esophageal pressure measurement in patients with respiratory failure. Am J Respir Crit Care Med. (2014) 189:520–31. doi: 10.1164/rccm.201312-2193CI, PMID: 24467647

[7] The PLeUral Pressure Working Group (PLUG—Acute Respiratory Failure section of the European Society of Intensive Care Medicine) Mauri T Yoshida T Bellani G Goligher EC Carteaux G. Esophageal and transpulmonary pressure in the clinical setting: meaning, usefulness and perspectives. Intensive Care Med. (2016) 42:1360–73. doi: 10.1007/s00134-016-4400-x, PMID: 27334266

[8] Perez J Dorado JH Papazian AC Berastegui M Gilgado DI Cardoso GP. Titration and characteristics of pressure-support ventilation use in Argentina: an online cross-sectional survey study. Rev Bras Ter Intensiva. (2020) 32:81–91. doi: 10.5935/0103-507X.20200013 32401994 PMC7206961

[9] Al-Bassam W Dade F Bailey M Eastwood G Osawa E Eyeington C. "likely over-assistance" during invasive pressure support ventilation in patients in the intensive care unit: a multicentre prospective observational study. Crit Care Resusc. (2019) 21:18–31. doi: 10.1016/S1441-2772(23)00572-0, PMID: 30857508

[10] Miao MY Chen W Zhou YM Gao R Song DJ Wang SP. Validation of the flow index to detect low inspiratory effort during pressure support ventilation. Ann Intensive Care. (2022) 12:89. doi: 10.1186/s13613-022-01063-z, PMID: 36161543 PMC9510081

[11] Bellani G Grassi A Sosio S Foti G. Plateau and driving pressure in the presence of spontaneous breathing. Intensive Care Med. (2019) 45:97–8. doi: 10.1007/s00134-018-5311-9, PMID: 30006893

[12] Foti G Cereda M Banfi G Pelosi P Fumagalli R Pesenti A. End-inspiratory airway occlusion: a method to assess the pressure developed by inspiratory muscles in patients with acute lung injury undergoing pressure support. Am J Respir Crit Care Med. (1997) 156:1210–6. doi: 10.1164/ajrccm.156.4.96-02031 9351624

[13] Kyogoku M Shimatani T Hotz JC Newth CJL Bellani G Takeuchi M. Direction and magnitude of change in plateau from peak pressure during inspiratory holds can identify the degree of spontaneous effort and elastic workload in ventilated patients. Crit Care Med. (2020) 49:517–26. doi: 10.1097/CCM.0000000000004746 PMC8176786 33252373

[14] Yang YL Liu Y Gao R Song DJ Zhou YM Miao MY. Use of airway pressure-based indices to detect high and low inspiratory effort during pressure support ventilation: a diagnostic accuracy study. Ann Intensive Care. (2023) 13:111. doi: 10.1186/s13613-023-01209-7, PMID: 37955842 PMC10643759

[15] Cohen JF Korevaar DA Altman DG Bruns DE Gatsonis CA Hooft L. STARD 2015 guidelines for reporting diagnostic accuracy studies: explanation and elaboration. BMJ Open. (2016) 6:e012799. doi: 10.1136/bmjopen-2016-012799, PMID: 28137831 PMC5128957

[16] Luo XY He X Zhou YM Wang YM Chen JR Chen GQ. Patient-ventilator asynchrony in acute brain-injured patients: a prospective observational study. Ann Intensive Care. (2020) 10:144. doi: 10.1186/s13613-020-00763-8, PMID: 33074406 PMC7570406

[17] Baydur A Behrakis PK Zin WA Jaeger M Milic-Emili J. A simple method for assessing the validity of the esophageal balloon technique. Am Rev Respir Dis. (1982) 126:788–91. PMID: 7149443 10.1164/arrd.1982.126.5.788

[18] Bianchi I Grassi A Pham T Telias I Teggia Droghi M Vieira F. Reliability of plateau pressure during patient-triggered assisted ventilation. Analysis of a multicentre database. J Crit Care. (2022) 68:96–103. doi: 10.1016/j.jcrc.2021.12.002 34952477

[19] Bellani G Grassi A Sosio S Gatti S Kavanagh BP Pesenti A. Driving pressure is associated with outcome during assisted ventilation in acute respiratory distress syndrome. Anesthesiology. (2019) 131:594–604. doi: 10.1097/ALN.0000000000002846, PMID: 31335543

[20] Sassoon CS Light RW Lodia R Sieck GC Mahutte CK. Pressure-time product during continuous positive airway pressure, pressure support ventilation, and T-piece during weaning from mechanical ventilation. Am Rev Respir Dis. (1991) 143:469–75. doi: 10.1164/ajrccm/143.3.469, PMID: 2001053

[21] Bertoni M Telias I Urner M Long M Del Sorbo L Fan E. A novel non-invasive method to detect excessively high respiratory effort and dynamic transpulmonary driving pressure during mechanical ventilation. Crit Care. (2019) 23:346. doi: 10.1186/s13054-019-2617-0, PMID: 31694692 PMC6836358

[22] de Vries HJ Tuinman PR Jonkman AH Liu L Qiu H Girbes ARJ. Performance of noninvasive airway occlusion maneuvers to assess lung stress and diaphragm effort in mechanically ventilated critically ill patients. Anesthesiology. (2023) 138:274–88. doi: 10.1097/ALN.0000000000004467, PMID: 36520507

[23] Albani F Fusina F Ciabatti G Pisani L Lippolis V Franceschetti ME. Flow index accurately identifies breaths with low or high inspiratory effort during pressure support ventilation. Crit Care. (2021) 25:427. doi: 10.1186/s13054-021-03855-4, PMID: 34911541 PMC8672539

[24] Albani F Pisani L Ciabatti G Fusina F Buizza B Granato A. Flow index: a novel, non-invasive, continuous, quantitative method to evaluate patient inspiratory effort during pressure support ventilation. Crit Care. (2021) 25:196. doi: 10.1186/s13054-021-03624-3, PMID: 34099028 PMC8182360

[25] Rittayamai N Beloncle F Goligher EC Chen L Mancebo J Richard JM. Effect of inspiratory synchronization during pressure-controlled ventilation on lung distension and inspiratory effort. Ann Intensive Care. (2017) 7:100. doi: 10.1186/s13613-017-0324-z, PMID: 28986852 PMC5630544

[26] Telias I Junhasavasdikul D Rittayamai N Piquilloud L Chen L Ferguson ND. Airway occlusion pressure as an estimate of respiratory drive and inspiratory effort during assisted ventilation. Am J Respir Crit Care Med. (2020) 201:1086–98. doi: 10.1164/rccm.201907-1425OC, PMID: 32097569

[27] Roesthuis L van den Berg M van der Hoeven H. Non-invasive method to detect high respiratory effort and transpulmonary driving pressures in COVID-19 patients during mechanical ventilation. Ann Intensive Care. (2021) 11:26. doi: 10.1186/s13613-021-00821-9, PMID: 33555520 PMC7868882

[28] Soundoulounaki S Akoumianaki E Kondili E Pediaditis E Prinianakis G Vaporidi K. Airway pressure morphology and respiratory muscle activity during end-inspiratory occlusions in pressure support ventilation. Crit Care. (2020) 24:467. doi: 10.1186/s13054-020-03169-x, PMID: 32723356 PMC7385937

[29] Docci M Rezoagli E Teggia-Droghi M Coppadoro A Pozzi M Grassi A. Individual response in patient's effort and driving pressure to variations in assistance during pressure support ventilation. Ann Intensive Care. (2023) 13:132. doi: 10.1186/s13613-023-01231-9, PMID: 38123757 PMC10733248

[30] Putensen C Muders T Varelmann D Wrigge H. The impact of spontaneous breathing during mechanical ventilation. Curr Opin Crit Care. (2006) 12:13–8. doi: 10.1097/01.ccx.0000198994.37319.60 16394778

[31] Yang J Zhang B Chaomin H Jiang X Shui P Huang J. Identification of clinical subphenotypes of sepsis after laparoscopic surgery. Laparosc Endosc Robot Surg. (2024) 7:16–26. doi: 10.1016/j.lers.2024.02.001

